# Targeted treatment in complex lymphatic anomaly: a case of synergistic efficacy of trametinib and sirolimus

**DOI:** 10.1186/s13023-024-03211-z

**Published:** 2024-05-16

**Authors:** Emmanuel Seront, Antoine Froidure, Nicole Revencu, Valerie Dekeuleneer, Philippe Clapuyt, Dana Dumitriu, Miikka Vikkula, Laurence M. Boon

**Affiliations:** 1 Institut Roi Albert II, Department of Medical Oncology, Center for Vascular Anomalies, Saint-Luc University Hospital, VASCERN VASCA European Reference Centre, UCLouvain, Brussels, Belgium; 2 Department of Pneumology, Center for Vascular Anomalies, Saint-Luc University Hospital, VASCERN VASCA European Reference Centre, UCLouvain, Brussels, Belgium; 3 Center for Human Genetics, Center for Vascular Anomalies, Saint-Luc University Hospital, VASCERN VASCA European Reference Centre, UCLouvain, Brussels, Belgium; 4 Division of Plastic Surgery, Center for Vascular Anomalies, Saint-Luc University Hospital, VASCERN VASCA European Reference Centre, UCLouvain, Cliniques Universitaires St Luc, Avenue Hippocrate 10, Brussels, B-1200 Belgium; 5 Present Address: Department of Pediatric Radiology, Center for Vascular Anomalies, Saint-Luc University Hospital, VASCERN VASCA European Reference Centre, UCLouvain, Brussels, Belgium; 6 Human Molecular Genetics, De Duve Institute, UCLouvain, Brussels, Belgium; 7 WELBIO Department, WEL Research Institute, Avenue Pasteur, 6, Wavre, 1300 Belgium

**Keywords:** Complex lymphatic anomaly, Sirolimus, Trametinib, VASE, Lymphedema, Infection

## Abstract

Repurposing anticancer drugs to vascular malformations has significantly improved patient outcomes. Complex Lymphatic Anomalies (CLA) are part of the spectrum of lymphatic malformations (LMs) that share similar oncogenic mutations to cancer. We report the case of a young patient with highly symptomatic CLA who was initially treated with sirolimus, due to the frequent involvement of the PI3K-AKT-mTOR pathway in CLA pathogenesis. Despite an initial reduction in symptoms, sirolimus progressively lost its effectiveness. After an unsuccessful attempt with trametinib alone, sirolimus was added to trametinib and resulted in a significant, rapid and sustained improvement in symptoms. This suggests that, contrary to current dogmas, combination therapy using sub-therapeutic doses targeting both the PI3K and RAS pathways retains efficacy without generating the toxicity known for combination therapies, and is beneficial in the management of CLAs and potentially other vascular anomalies.

**Supplementary Information:**

The online version contains supplementary material available at 10.1186/s13023-024-03211-z.

## Introduction

Lymphatic malformations (LMs) result from anomalies in the development of lymphatic vessels (lymphangiogenesis), leading to congenital malformations that evolve with the patient. In the 2018 classification of the International Society for the Study of Vascular Anomalies, LMs are subdivided into common (cystic) LMs and a variety of other conditions called Complex Lymphatic Anomalies (CLAs). The latter mainly include generalized lymphatic anomaly (GLA), Kaposiform Lymphangiomatosis (KLA), Gorham-Stout disease (GSD) and central conducting lymphatic anomalies (CCLAs) [1, 2].

CLAs are rare diseases that involve multiple localizations and result in disturbed fluid homeostasis fluid accumulation (chylous ascites, chylothorax, chyluria), malnutrition due to enteropathy and potential immune dysfunction [1–4]. There is considerable phenotypic heterogeneity with overlapping symptoms, aspecific imaging features and complications. Thus, the management of CLAs remains challenging, and requires a multimodal approach [5].

Two important signaling pathways are involved in lymphangiogenesis, regulating the growth, proliferation and survival of lymphatic endothelial cells: the phospho-inositol 3 kinase (PI3K)—Protein B (AKT)—mammalian target of Rapamycin (mTOR)—cascade and the Mitogen Activated Protein Kinase (MAPK) cascade. Alterations in these pathways, which are share similarities with cancer, can lead to the development of LMs and CLAs. The *PIK3CA* activating mutation*,* which leads to constitutive activation of AKT by PI3K and sustained mTOR activation, is found in up to 80% of LMs. The presence of *PIK3CA*-activating somatic mutations has also been detected in some GLA, KLA and CCLA patients [6, 7]. The mTOR inhibitor sirolimus significantly improved the outcome in some CLA patients, including those in the prospective phase III VASE trial (EudraCT 2015-001703-32). Response to sirolimus ranged from 50 to 100% depending on the trial and population [8, 9].

Mutations in genes encoding components of the RAS-MAPK pathway have also been identified in CLA patients. These mutations, including activating mutations of *NRAS*, *KRAS*, *ARAF*, and an inactivating mutation of *CBL*, lead to activation of the MAPK pathway, resulting in excessive ERK activation. While the efficacy of trametinib monotherapy was reported in some case reports on patients with CLAs [10–14], no report has yet demonstrated feasibility of combining sirolimus plus trametinib.

We report for the first time the effective management of CLA with low-dose combination therapy of sirolimus and trametinib, after failure of mTOR and MEK inhibitors alone, without increasing the toxicity profile.

## Case report

In 2017, a 16-year-old patient was highly symptomatic and referred to our multidisciplinary Center for Vascular Anomaly, with a diagnosis of CLA.

**Fig. 1:**
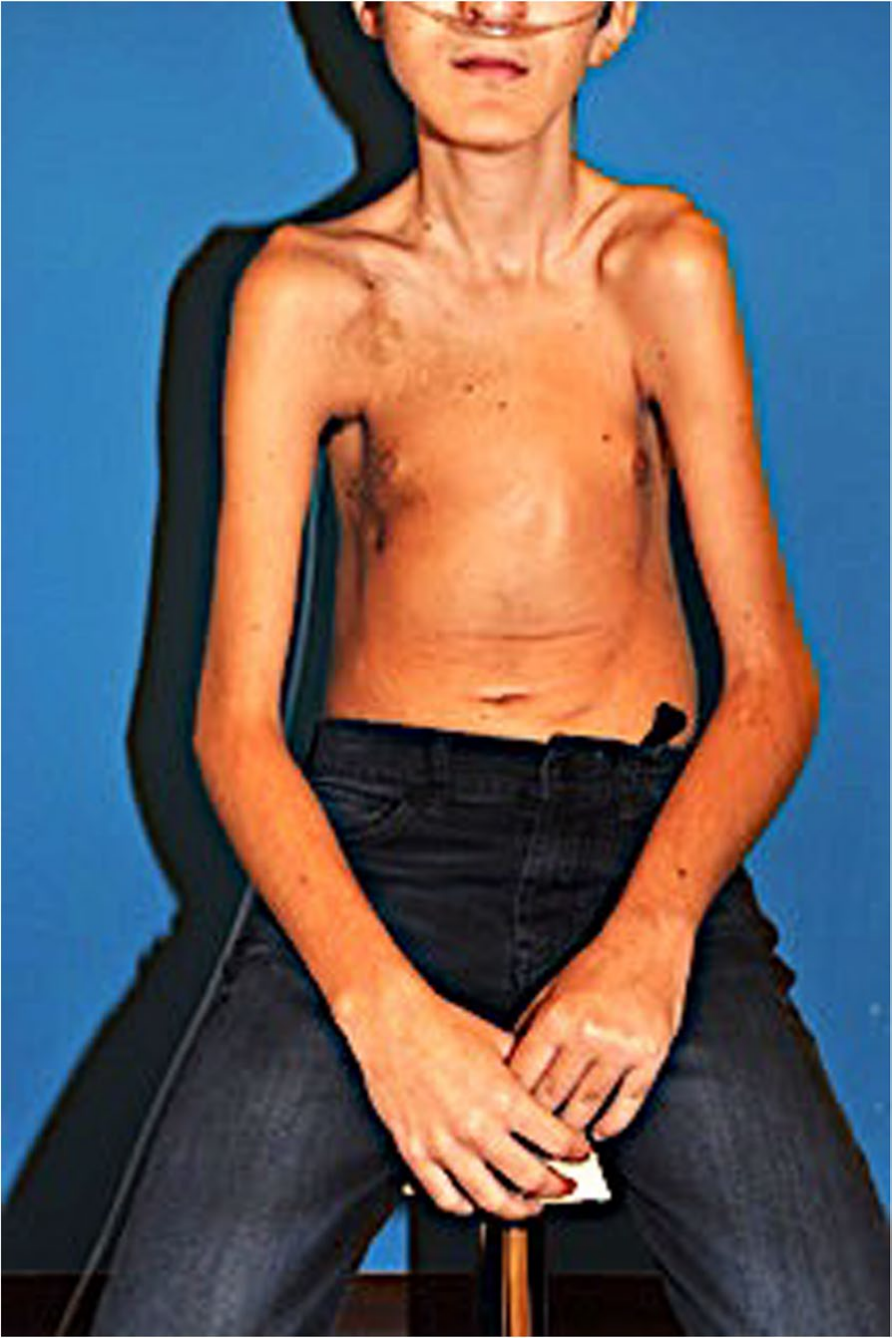
Restrictive pulmonary syndrome, associated with scoliotic deformity, respiratory muscle atrophy, and growth restriction

During the first years of life, the patient suffered multiple respiratory infections (viral and bacterial), associated with progressive growth retardation. In 2009, at the age of 8, he developed lymphedema of the left arm and progressive dyspnea. Imaging revealed bone lysis (4th left rib and scapula), bilateral pleural effusions, and pulmonary infiltrations. After multiple pleurocentesis to remove abundant chyle, a thoracic duct clipping was performed and a pleuroperitoneal drain was placed. On the basis of suspected diagnosis of Gorham-Stout disease, interferon-based systemic treatment was started in 2009 for a period of 6 months. BiPAP nocturnal support and oxygenotherapy were also introduced. Gradually, the patient developed a restrictive pulmonary syndrome, aggravated by scoliotic deformity and respiratory muscle atrophy (Fig. 1). Dyspnea increased over time, even at rest, necessitating an increase in continuous supply of oxygen (24 h per day). In 2010, due to an episode of severe pneumonia, the patient underwent a single course of intravenous ImmunoGlobulin (IVIG) treatment. In 2015, due to continuous deterioration in pulmonary status, bevacizumab (intravenous, three courses) was initiated with no clinical or pulmonary improvement.

**Fig. 2:**
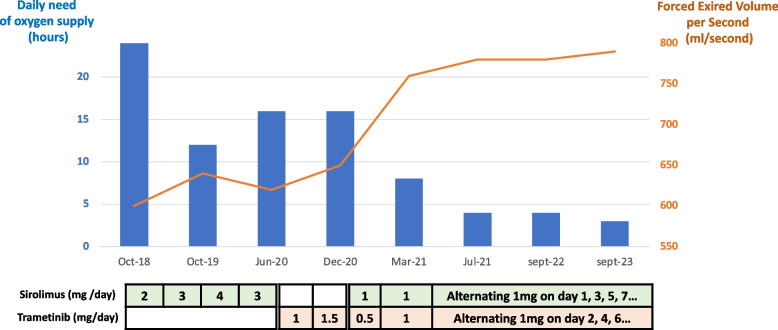
Evolution of daily oxygen supply and FEVS on different treatment regimens. Dosages given for sirolimus and trametinib monotherapy, and combination therapy

**Fig. 3:**
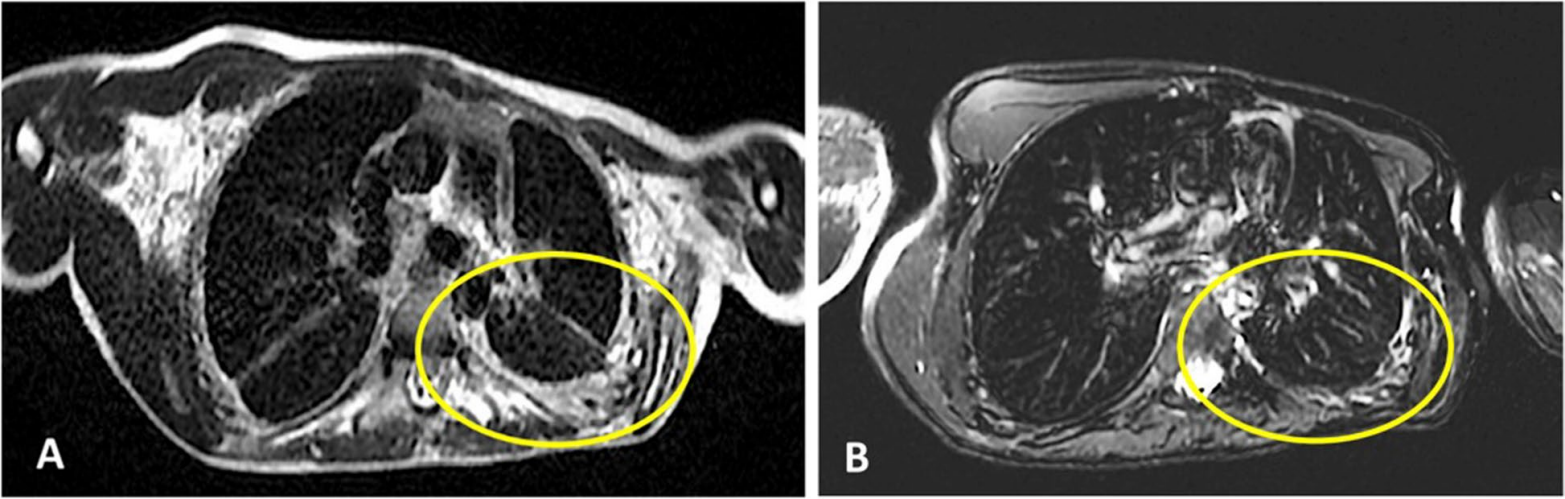
**A** Magnetic Resonance Imaging (MRI-T2) of thorax in 2018 (before starting sirolimus): pleural thickening (yellow circle). **B** MRI of thorax in 2022 (on sirolimus + trametinib): reduction of pleural thickening

At the time of our first consultation in 2017, he was presenting with persistent grade 4 dyspnea, as well as daily headache and recurrent pulmonary infections (3-4 episodes per year). He was completely dependent on oxygen, with 2 L/min, 24 h a day (with BiPAP for 8 h at night) and 3 L/min for exercice, such as walking 100 m to catch the bus to school 5 days a week. He was unable to do any other effort or practice any sport. The patient was cachectic, with a body mass index of 14 (Fig. 1). Forced Expired volume in one second (FEVS) was 600 mL (22% of predicted values), Forced Vital Capacity (FVC) was 600 mL (15% of predicted values) and Total Lung Capacity (TLC) was 1.690 L (40% of predicted values) (Fig. 2). Blood tests were within normal range, including the red blood cell count, leucocyte profile and liver and kidney function. Magnetic resonance imaging (MRI) of the chest showed pleural thickening (Fig. 3A).

Due to recurrent pulmonary episodes, we started sirolimus monotherapy in November 2018 with a dosage ranging from 2 to 4 mg daily to achieve a serum sirolimus level between 10 and 15 ng/ml (Fig. 2). Sirolimus was well tolerated, with intermittent grade 2 mucositis well managed with mouthwashes, grade 1 asthenia and intermittent grade 1 diarrhea (Common Terminology Cancer Adverse Events; CTCAE version 4.0). On sirolimus, lymphoedema in the left-arm began to diminish over the first 3 months, and subjective sensation of dyspnea and headache intensity and frequency progressively decreased, as did oxygen intake (2 L/min, 12 h instead of 24 h per day; but persistence of 3 L/min when walking to catch the bus 5 days a week). However, he was still unable to make any other effort or take part in any other sports. Lung function remained stable after 12 months of sirolimus: FEVS was 640 mL, FVC 620 mL and TLC 2.0 L. He had no pulmonary infections during the 12 months of sirolimus treatment (Fig. 2).

After this timepoint, the clinical condition deteriorated slightly, with an increase in the sensation of dyspnea at rest, an increase in the frequency of headaches, an increased need for oxygen intake (2 L/min 16-18 h daily, with a persistent 3 L/min for walking 100 m) and the resurgence of an episode of a pulmonary infection. The patient himself reported that the efficacy of sirolimus seemed to be diminishing, despite correct serum levels. FEVS decreased slighty compared to previous values (620 mL/sec).

In July 2020, we decided to stop sirolimus and start trametinib with a daily dose of 1 to 1.5 mg. The patient described a slight clinical improvement in the first three months, although without a decrease in oxygen intake. Gradually, this benefit disappeared, with a re-increase in the sensation of dyspnea, headache frequency/intensity and persistent pulmonary infection (two courses of amoxycilline). Trametinib was associated with grade 1 acneiform rash and grade 1 asthenia. Six months after trametinib initiation, there was no improvement in pulmonary function (FEVS 650 mL/sec. Despite acceptable tolerance, the patient reported a better quality of life and a greater clinical improvement with sirolimus compared to trametinib (Fig. 2).

In December 2020, we added sirolimus to trametinib, starting with a daily dose of 1 mg sirolimus and 0.5 mg trametinib. Tolerance was good, allowing us to increase the daily dose to 1 mg for both drugs after 2 weeks. This regimen resulted in significant clinical improvement over the first three months (Fig. 2). The patient experienced a reduction in dyspnea and increased in daily activities with his friends, including walking (three times per week). Oxygen intake significantly decreased (2 L/min, 8 h a day, with no systemic need for oxygen to walk the 100 m to the bus). He became able to work part-time as an administrative consultant. He still needed BiPAP assistance at night. The headaches and pulmonary infections have disappeared. In addition, FEVS increased gradually over three months, reaching 760 mL/sec (Fig. 2).

This efficacious combination treatment was associated with adverse events, including grade 2 asthenia, grade 2 diarrhea and grade 2 mucositis. We adapted the regimen after two months, alternating the daily doses of 1 mg of sirolimus and 1 mg of trametinib. The dose adjustement resulted in a better tolerance with only intermittent grade 1 diarrhea.

The benefit after six months of the sirolimus-trametinib combination treatment (three months with the alternate daily regimen) was evident, with sustained clinical improvement, including disappearance of headaches, increased daily activities, decreased daily oxygen requirement, and increased FEVS (reaching 780 mL/sec), as well as an increase in body mass index that reached 17. He was able to do some sports, such as cycling (once per week), rower (twice per week), elliptical bike (twice per week). He is now working full-time. Our patient has been on this treatment for 3 years, with no recurrence of lung infections, and stable clinical and pulmonary function. MRI of the chest performed in 2022 showed a decrease in pleural thickening, which had been observed in 2017 before starting sirolimus treatment (Fig. 3A and B). No MRI was performed between 2018 and 2022 due to the difficulty for the patient to perform such type of imaging.

## Discussion

This case report highlights for the first time the potential efficacy and feasibility of combining sirolimus and trametinib as a treatment regimen for a patient with a highly symptomatic CLA. Despite the inability to perform tissular genetic testing to identify the patients’ eventual somatic mutation, the decision to administer this combination therapy was based on the current knowledge on the pathophysiological bases of vascular anomalies and CLAs. *PIK3CA* mutations are observed in around 80% of LMs, and are also implicated in some CLAs [15]. Therefore, the initial treatment approach for this patient was sirolimus (up to 4 mg/day) for a duration of 18 months. Despite clinical improvement, including reduced symptoms, reduced reliance on oxygen supplementation and fewer episodes of infection, sirolimus did not significantly improve lung function. Sirolimus progressively lost its efficacy over time, reflecting potential activation of alternative, interconnected pathways. Given that several CLA patients have activating mutations in genes encoding members of the RAS-RAF-MEK1/2-ERK pathway, trametinib, a MEK inhibitor was investigated as potential treatment option. However, its administration did not significantly improve the clinical or pulmonary status of the patient, suggesting that the RAS-RAF-MEK cascade was not the primary driver of the CLA in this instance.

Activation of mTORC1 has been shown to lead to inhibition of both PI3K and MAPK via a negative feedback loop originating from S6 kinase, a downstream effector of mTOR. Consequently, treatment with mTORC1 inhibitors may increase activation of the RAS-RAF-MEK1/2-ERK pathway [16]. Furthermore, the majority of CLAs appear to harbor a mutation that activates the RAS pathway [17]. Notably, KRAS can activate PI3K, while PI3K can also activate RAS, indicating crosstalk between these two pathways. In light of these concepts, the decision was made to test the combination of sirolimus with trametinib, aiming to concurrently inhibit both pathways. This combination led to notably improved pulmonary function and quality of life for the patient. These findings underscore the importance of understanding the intermingled play between these two signaling pathways: the PI3K-AKT-mTor and the RAS-RAF-MEK1/2-ERK pathways.

The combination of sirolimus 1 mg and trametinib 1 mg demonstrated rapid and significant efficacy in treating our patient. However, it was associated with higher toxicity, as expected. The long half-life of sirolimus (approximately 62 h) and trametinib (approximately 120 h) enabled us to further adjust dosing. Specifically, each medication was administered every second day, reducing the daily intake by 50%, reducing potential side effects. Remarkably, this modified dosing schedule did not compromise the treatment efficacy. Conversely, the incidence and severity of adverse effects decreased substantially, enhancing the patient’s quality of life. This approach aligns with the finding of the VASE study, as well as the thalidomide regimen for arteriovenous malformations; both have demonstrated that once an effective concentration is achieved, subsequent maintenance regimens can use reduced dosages to diminish side effects without affecting therapeutic efficacy [9, 18]. This is likely even more so for vascular anomaly treatments that target in parallel the two interconnected pathogenic signaling pathways.

Sirolimus was initially considered as an immunosuppressive agent. Yet, during sirolimus monotherapy, the frequency and intensity of pulmonary infections of the patient were reduced. This might be due to improved pulmonary function or better pulmonary diffusion of immune cells through increased lymphatic drainage. Anyhow, no sign of immunosuppression was seen. Furthermore, this patient was vaccinated against COVID-19 while on sirolimus treatment and developed serum antibodies, confirming the absence of immune decrease induced by sirolimus.

## Conclusion

This case report introduces novel possibilities for the management of patients with complex lymphatic anomalies (CLAs) with likely underlying activation of the PI3K-AKT-mTOR and the RAS-RAF-MEK1/2-ERK pathways. The data suggest that the use of low doses of mTOR and MEK inhibitors in combination may improve their efficacy over monotherapy, without increasing the toxicity profile. This offers hope for a brighter future for these patients with unmet medical needs. Clinical trials and subsequent management guidelines are naturally needed.

### Supplementary Information

Supplementary Material 1.

## Data Availability

Materials described in the manuscript (spirometry results) are freely available to any scientist in the supplementary files (Sirotrame.xls), without reaching participant confidentiality.
